# 

**Published:** 1957-04

**Authors:** 


					v 'f~<? //
1 V:-/
THE
medical journal
OF THE
SOUTH-WEST
Incorporating
The Bristol Medico-Chirurgical Journal
April 1957
VOL. 7
1 00- No. 264 Price 4/'

				

